# Co-culture of dendritic cells and cytokine-induced killer cells effectively suppresses liver cancer stem cell growth by inhibiting pathways in the immune system

**DOI:** 10.1186/s12885-018-4871-y

**Published:** 2018-10-16

**Authors:** Tao Yang, Wenjun Zhang, Li Wang, Chunyan Xiao, Li Wang, Yi Gong, Dehong Huang, Bingling Guo, Qiying Li, Ying Xiang, Yingyu Nan

**Affiliations:** 1 Chongqing Key Laboratory of Translational Research for Cancer Metastasis and Individualized Treatment, Chongqing University Cancer Hospital & Chongqing Cancer Institute & Chongqing Cancer Hospital, No.181 Hanyu Road, Chongqing, 400030 People’s Republic of China; 2 Department of Oncology, Chongqing General Hospital, Chongqing, People’s Republic of China

**Keywords:** Dendritic cells, Cytokine-induced killer cells, Hepatocellular carcinoma, Apoptosis, Caspase-3, Liver cancer stem cells

## Abstract

**Background:**

Application of dendritic cells (DC) for cancer immunotherapy involves tumor-associated immunogenic antigens for effective therapeutic strategies. The present study investigated whether DC co-cultured with autologous cytokine-induced killer cells (CIK) could induce a more specific immune response against liver cancer stem cells (LCSC) generated from human hepatocellular carcinoma (HCC) cells in vitro and in vivo.

**Methods:**

Human DC and CIK were generated from peripheral blood mononuclear cells (PBMCs) taken from consenting liver cancer patients. Flow cytometry was used to determine the phenotypes of DC and CIK, and cell proliferation. The tumor growth and anti-tumor activity of these cells were further evaluated using a nude mouse tumor model.

**Results:**

We demonstrated that DC and CIK significantly enhanced the apoptosis ratio, depending on DC-CIK cell numbers, by increasing caspase-3 protein expression and reducing proliferating cell nuclear antigen (PCNA) protein expression against LCSC. The in vivo data indicated that DC-CIK exhibited significant LCSC cell-induced tumor growth inhibition in nude mice, which was most significant with LCSC antigen loaded DCs.

**Conclusions:**

The results showed, that DC-CIK cells could inhibit HCC and LCSC growths in vitro and in vivo and the most successful DC triggering of cell cytotoxic activity could be achieved by their LCSC antigen loading.

## Background

Cancer stem cells (CSC) have recently been considered to be present in malignant tumors and are characterized by infinite proliferation, self-renewal and a multi-directional differentiation capacity [1]. By targeting the markers cluster of differentiation (CD) 90 [2], CD44 [3], CD133 [4] and epithelial cell adhesion molecule (EpCAM) [5], it has proven possible to differentiate between liver cancer stem cells (LCSC) and liver cancer cells. This research suggests that more attention should be paid to LCSC treatment in clinical liver cancer therapy. The presence of LCSC is likely to induce resistance to chemotherapy and recurrence in liver cancer cells [6, 7]. Therefore, how to treat LCSC must be considered after an operation, radiotherapy and/or chemotherapy. Autoimmune treatment of a malignant tumor is considered to be a feasible method, that mainly depends on the interfering and suppressing effects of killer cells induced by the tumor, infiltrating lymphocytes and lymphokines, as well as CD3 monoclonal antibodies [8, 9]. Currently, cytokine-induced killer cells (CIK) therapy or dendritic cells (DC)-CIK cell co-cultivation has been widely used to treat malignant tumors in clinical trials, because DC and CIK have been demonstrated to possess high antitumor and cytotoxic activity against hepatocellular carcinoma (HCC) cells in vitro and in vivo [10–12]. DCs with their antigen-presenting ability make them attractive vehicles for therapeutic tumor vaccine delivery and for providing a vaccine development platform [13]. CIK were obtained from human peripheral blood mononuclear cells (PBMCs) stimulated by recombinant human interferon gamma (rhIFN-γ), interleukin (IL)-2 and CD monoclonal antibodies. and express surface markers of T cells and natural killer (NK) cells [14]. The CIK possessing the ability to attack tumor cells expressing CD3/CD56 on the cell surface have antitumor activity against a variety of cancer types, particularly when co-cultured with antigen-loaded DCs. Although there are several reports about DC and CIK therapies, the mechanism of effective inhibition of LCSC by DC and CIK cells remains unclear. Therefore, this research established a nude mouse LCSC tumorigenic model and hypothesized that the inhibitory effect of DC-CIK co-culture on LCSC is caused by suppressing proliferating cell nuclear antigen (PCNA) and increasing the caspase-3 pathway. In addition, we demonstrated a DC printing relationship between DC-CIK numbers and the degree of LCSC induced tumor suppression.

## Methods

### Patients

Seven cases of advanced liver cancer patients (18–75 years old) were enrolled in the study, who were treated in the Chongqing Tumor Research Institution from June 2013 to March 2014. Routine blood tests and functions did not show other underlying diseases of the heart or kidney. All patients were shown to have stage III or IV liver cancer through histological analysis. There were 5 cases of measurable lesions and 2 cases of immeasurable lesions (including pleural and peritoneal effusion). Their last treatments including surgery, radiotherapy and chemotherapy was more than one month ago and their life expectancies were greater than 3 months. The patients were not willing to undergo, or were inappropriate for, other treatments (surgery, radiotherapy, chemotherapy). The Karnofsky Performance Status scores were > 60 points. These patients had not previously received any autologous cell immune therapy. All the patients or a legal representative signed informed consent forms and this experiment was approved by the ethics committee of the Chongqing Tumor Research Institution.. After evaluating the curative effect, all patients had a follow up visit once every 3 months for corresponding imaging and blood tests. The follow up period was from September 2013 to September 2014.

### Animals

Twenty-seven nude mice (6–8 weeks old) were purchased from the animal center of the Third Military Medical University (Chongqing, China). The mice were housed under specific pathogen-free conditions. All the experiments were performed according to the National Institutes of Health Guide for Care and Use of Laboratory Animals (National Institutes of Health, Bethesda, MD, USA) and were approved by the Bioethics Committee of the Third Military Medical University.

### Human cell lines

All used cell lines were controlled with a mycoplasma detection kit (Plasmo Test kit, rep-pt1, InvivoGen, California USA) to confirm that the cells were mycoplasma contamination free before they were used for further studies.

The human HCC cell lines HepG2 (ATCC HB-8065) and SMMC7721 cells (CCTCC GDC064) were purchased from the American Type Culture Collection (ATCC; Rockville, MD, USA) and China Center for Type Culture Collection (CCTCC), respectively and maintained in our laboratory. The cells were cultured in complete RPMI 1640 medium (RPMI 1640, Invitrogen Life Technologies, Carlsbad, CA, USA) and supplemented with 10% heat-inactivated fetal bovine serum (Gibco Life Technologies, Carlsbad, CA, USA), 100 U/ml penicillin and 100 mg/ml streptomycin (Sigma-Aldrich, St. Louis, MO, USA) at 37 °C in a 5% CO_2_ atmosphere.

### Generation of LCSCs from HepG2 and SMMC7721 cells

After logarithmic growth of HepG2 and SMMC7221 cells, they were transferred onto ultralow adhere culture plates (Cat. No. 3412, Constar, Corning Incorporated, USA) with stem cell culture medium to induce LCSC suspension clusters, which was exchanged by 50% every 2.5 days. The stem cell induction medium consisted of serum-free medium (SFM) (Cat. No. 12-725f, Lonza) with leukemia inhibitory factor (LIF) (Cat. No. CYT-644, ProSpec-Tany, TechnoGene, Ltd.) (20 ng/ml), epidermal growth factor (EGF) (Cat. No. 01–107, Millipore) (20 ng /ml), basic fibroblast growth factor (bFGF) (Cat. No. GF003, Millipore) (20 ng/ml), 2% B27 (Cat. No. 17504–044, Gibco), streptomycin (100 U/ml) and penicillin (100 U/ml). The growth status of LCSC cell-clusters was monitored daily. The induced cells were incubated for 1 h at 37 °C in darkness with Anti-Human CD90 (Thy-1) FITC (Cat. No. 11–0909-41, eBioscience) and Anti-Human CD133/1 (AC133)-PE (Cat. No. 130–080-801, Miltenyibiotec) antibodies. After resuspension into PBS, the percentages of antibody labeled cells were detected by flow cytometry (BD FACS Calibur, Franklin Lakes, New Jersey, USA).

### Generation of DC and CIK

DC and CIK were generated from PBMCs taken from consenting advanced hepatic carcinoma patient volunteers according to the protocol approved by the ethics committee of Chongqing Tumor Research Institution. DC and CIK were generated as previously described [15]. Briefly, PBMCs were isolated from whole blood by Ficoll density gradient centrifugation using a commercially available lymphocyte separation medium (Sigma-Aldrich) and centrifuged at 400×g for 25 min (NEW 2–21). Subsequently, the cells were allowed to adhere to six-well plates (Corning Life Sciences Tewksbury, MA, USA) at a density of 5 × 10^6^ cells/ml for 2 h at 37 °C in complete RPMI 1640 medium. The adherent and non-adherent cells were collected to generate DC and CIK, respectively. To generate DC, the adherent cells were cultured in complete RPMI 1640 medium with 1000 U/ml recombinant human (rh) granulocyte-macrophage colony-stimulating factor and 500 U/ml recombinant human interleukin-4 (rhIL-4) (R&D Systems, Minneapolis, MN, USA) at 37 °C in a humidified atmosphere of 5% CO_2_; immature DCs were collected. The medium, along with the necessary cytokines, was replaced every 3 days. On day 6, a further 1000 U/ml of tumor necrosis factor α (TNF-α) was added to the DC cells to induce maturation. To generate CIK, the non-adherent PBMC were prepared and grown in complete RPMI 1640 medium with 1000 U/ml rhIFN-γ (R&D Systems, Minneapolis, MN, USA). After a 24 h incubation, 50 ng/ml mouse anti-human CD3 monoclonal antibody and 1000 U/ml IL-2 were added. The CIK were incubated at 37 °C in a humidified atmosphere of 5% CO_2_ and sub-cultured every 3 days with cytokine replenishment. Co-cultures of LCSCs and HepG2 cells with DC + CIK cells were carried out with LCSC cells kept in non-adherent and DC + CIK in adherent culture conditions in a transwell chamber.

### Determination of DC and CIK cells

DC and CIK cells cultured on day 1 and day 7 were collected. After washing with PBS and centrifugation, the DC cells were incubated with FITC-conjugated mouse anti-human CD14 (Clone M5E2, Cat. No. 561712, BD Pharmingen, US), CD83 (Clone HB15e, Cat. No. 560929, BD Pharmingen, US) and CD86 (Clone 2331 (FUN-1), Cat. No. 560958, BD Pharmingen, US) monoclonal antibodies and PE-labeled mouse anti-human HLA-DR monoclonal antibody (Clone G46–6, Cat. No. 560943, BD Pharmingen, US) for 20 min at room temperature. The CIK cells were incubated with FITC-conjugated mouse anti-human CD3 (Clone HIT3a, Cat. No. 561802, BD Pharmingen, US) and CD4 (Clone RPA-T4, Cat. No. 561005, BD Pharmingen, US) monoclonal antibodies and PE-labeled mouse anti-human CD8 (Clone RPA-T8, Cat. No. 561949 BD Pharmingen, US) and CD56 (Clone B159, Cat. No. 561903, BD Pharmingen, US) monoclonal antibodies for 20 min at room temperature. Then the DC and CIK cells were washed with PBS twice. Flow cytometry was used to determine the phenotypes of DC and CIK cells.

### Analysis of IFN-γ-secreting CIK

IFN-γ-secreting cells were detected on day 7 of the co-culture using intracellular staining and flow cytometry [16, 17]. Briefly, the CIK and DC-CIK were suspended in complete RPMI 1640 and stimulated for 4 h with 25 ng/ml phorbol 12-myristate 13-acetate, 1 μM ionomycin and 2 μM monensin (Sigma-Aldrich). After washing with PBS, the cells were stained with FITC-conjugated mouse anti-human CD3 monoclonal antibody (Clone HIT3a, Cat. No. 561802, BD Pharmingen, US) for 30 min at 4 °C, washed with PBS and then permeabilized with FACS permeabilizing solution (BD Pharmingen) for 10 min at room temperature. The samples were then incubated with PE-labelled mouse anti-human IFN-γ monoclonal antibody (Clone B27, Cat. No. 562016, BD Pharmingen, US) for 30 min at room temperature in the dark, washed with PBS, and analyzed by flow cytometry for IFN γ secreting CIK.

### CCK-8 measurement

The cell counting kit-8 (CCK8) (Toyobo Co., Ltd., Japan) was used according to the manufacturer’s instructions. Inoculated first passage LCSC or HepG2 cells were placed into 6-well plates at 1 × 10^6^ cells/ml. After normal cultivation for 24 h, inoculated same density DC-CIK cells were matched into transwell 6-well plates. Co-cultured DC-CIK with LCSC or HepG2 was assembled in the transwell plates. The single cultured LCSC were the normal control group, co-cultured at the same density but the non-induced HepG2 cells were the positive controls with pure media acting as the blank control. The 6-well plates were removed after co-culture for 24 h, 48 h and 72 h and digested by trypsin. Inoculates were placed into 96-well plates (3000 cells/150 μl/well) and CCK-8 solution (approximately 10 μl per well) was added, and cultivated for 4 h under the same conditions (vide supra). After vortexing for 20 s and suspension, the absorbance at 450 nm was measured. The dye consisted of a 2-(2-methoxy-4-nitrophenyl)-3-(4-nitrophenyl)-5-(2,4-disulfophenyl)-2H- tetrazolium monosodium salt (WST-8) reduction by NADH from living cells. Each experiment was repeated 3 times.

### Construction of antigen loaded DCs

3×10^6^ LCSC and HepG2 cells in PBS were fragmented by three CO_2_ freeze-thaw cycles, then centrifuged for 10 min at 600×g and the supernatant was used for incubation with DCs from the day 3 of DC maturation

### Intradermal antigen loaded DC-CIK cell injections and solid tumor growth observation

The animal experiments were conducted 3 times. Each time tumors were generated by subcutaneous inoculation with LCSC 2 × 10^6^ cells/ml in 0.2 ml of PBS into the left, right axillary regions or left, right sides of the groins of 9 nude mice. After tumor growth on day 14, the mice were randomly divided into 3 groups with similar tumor sizes. The first group served as the control group without any treatment (LCSC-control, *n* = 3). The other 2 groups were treated with LCSC antigen loaded DC-CIK cells (2 × 10^6^ f-LCSC-DC-CIK, *n* = 3) and HepG2 antigen loaded DC-CIK cells (2 × 10^6^ f-HepG2-DC-CIK, *n* = 3). The treatments consisted of the f-LCSC-DC-CIK and f-HepaG2-DC-CIK mixtures in 0.05 ml PBS at 14 days after initial LCSC applications into 4 tumor surrounding tissue spots of the nude mice. Finally, in addition to the tumor weights, the subcutaneous tumor volume was measured 14 days later on day 28 after tumor initiation using a caliper and volume estimated as follows: Tumor volume (mm^3^) = 0.5 × length × width^2^.

### Western blotting

LCSC co-cultured for 24 h and 48 h with different densities of DC or CIK cell were harvested. The total cell protein was extracted and SDS-PAGE analysis conducted after quantification to separate the proteins. The proteins were transferred from the gel to a nitrocellulose membrane and the membrane incubated with primary antibodies (sheep anti-human caspase-3 polyclonal antibody (sc-1225, Santa Cruz Biotechnology Inc., USA), dilution 1:700) or PCNA mouse monoclonal antibody (sc-25,280, Santa Cruz Biotechnology Inc., USA) (dilution 1:800) overnight at 4 °C. Then the membrane was incubated with secondary antibodies (horse radish peroxidase labelled donkey anti-sheep IgG (A0181, Byotime, Beijing, China), dilution 1:500; or sheep anti-mouse antibody (A0216, Byotime, Beijing, China) at room temperature for 3 h. Finally, the color intensity was measured using an enhanced chemiluminescence method using Image-pro plus software. GAPDH was used as the internal reference. The OD ratio of caspase-3 and PCNA/GADPH indicated the relative expression levels of PCNA or caspase-3.

### CIK quality

An inverted microscope was used to check CIK growth, proliferation and for any contamination. Before collecting CIK, fungi, bacteria cultivation and endotoxins tests were conducted to establish that these exogenous factors were all negative. The survival rate of CIK was > 97% and the recovery survival rate after being frozen was > 75%.

### Statistical analysis

Values are expressed as the mean ± standard deviations of the mean. Statistical analysis was performed using Student’s *t*-test or one-way analysis of variance. Statistical analyses were performed using SPSS 16.0 software (SPSS, Inc., Chicago, IL, USA). *P* ≤ 0.05 was considered to indicate a statistically significant difference.

## Results

### Phenotype characteristics of DC and CIK

**Fig. 1:**
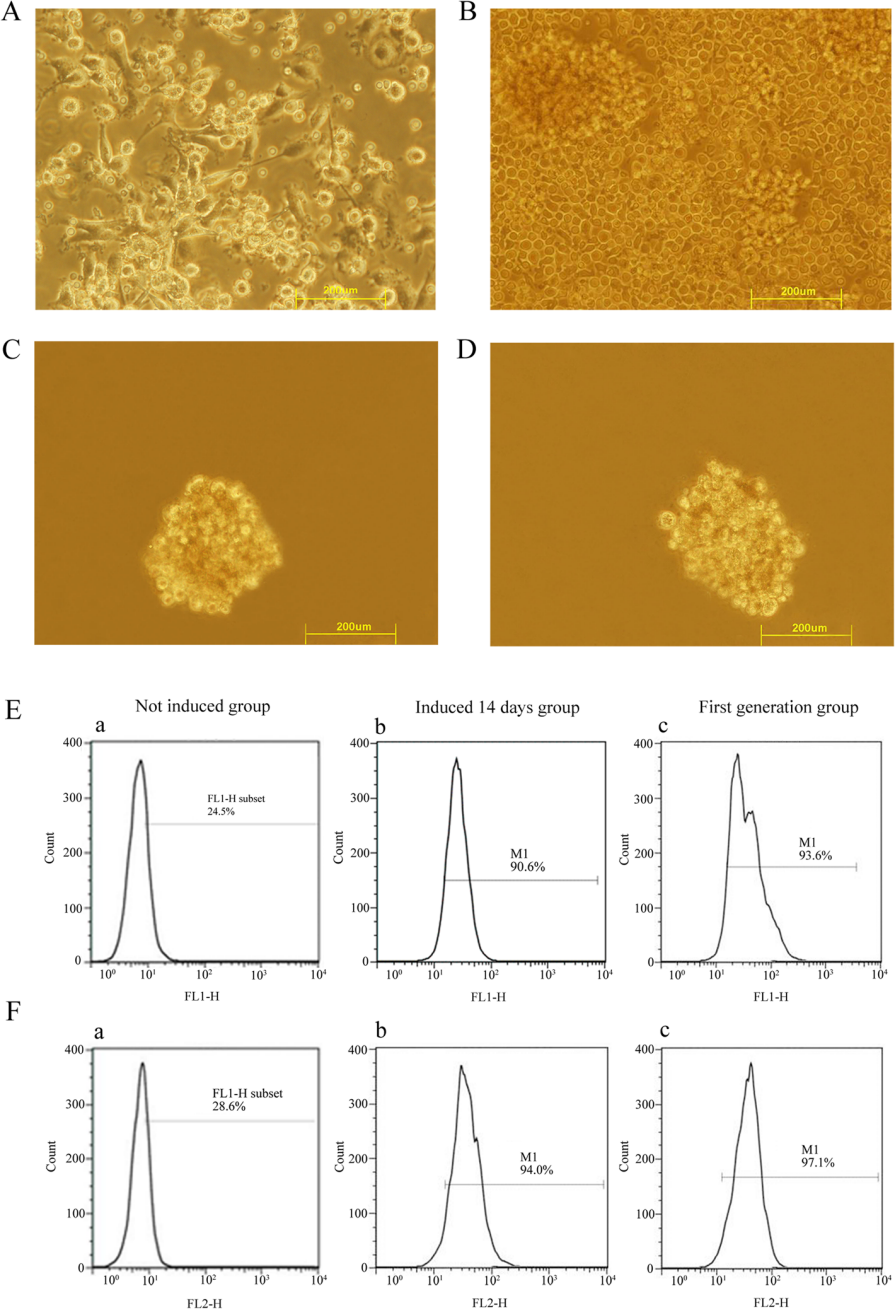
Morphology of DCs and CIK cells and LCSC induction and phenotypic characterization. **a** DCs formed dendritic shapes. **b** CIKs cells formed clusters. **c** First generation of LCSC from HepG2 cell. **d** First generation of LCSC from SMMC-7721 cell. **e** Flow cytometry analyses of the incidence of CD90 positivity. **f** Flow cytometry analyses of the incidence of CD133 positivity

According to a previous protocol [15], after 3–4 days cultivation, DC cells generated from PBMC formed different sizes of clones. The cell body changed to a circular or irregular shape and small dendritic pseudopodia stretched out from the cell membrane. With an extension of the culture time, dendritic protrusions on the surface increased to form the typical dendritic shape (Fig. 1a).

However, CIK cells generated from PBMC started to proliferate after being cultivated for 5 days. The cell bodies obviously increased at day 12, and the cells finally emerged irregularly shaped and grew in clusters (Fig. 1b). Thereafter, cells increased in numbers 2–3 times every 3 days and reached 13 times the inoculation density after 11 days. At this time, DC cells were added to start the co-culture (1 DC:10 CIK). The rate of cell increase became much faster, 2–3 times that when CIK were independently cultured.

### Phenotypic characterization of LCSC

LCSC cultures were derived from HepG2 and SMMC-7721 cells, consequently producing the first generation of LCSC.

The flow cytometry results indicated that the positive incidence of CD90 and CD133 for non-induced HepG2 cell was 24.5% (Fig. 1e-a) and 28.6% (Fig. 1f-a), respectively. The CD90 positive incidence of HepG2 cells induced for 14 days and the first generation were 90.6% (Fig. 1e-b) and 93.6% (Fig. 1e-c), respectively. The CD133 positive incidence of induced HepG2 cells was 94.0% (Fig. 1f-b) and 97.1% (Fig. 1f-c), respectively.

For the analysis of SMMC7721 cells, the positive incidence of CD90 and CD133 for the non-induced group was 33.9% and 36.1%, respectively. After induction, the positive incidence of CD90 and CD133 increased to 94.7% and 98.3% and for CD133 positive induction, the values were 94.5% and 99.4% (images not shown).

### Effects of DC-CIK on the cell cycle and apoptosis of LCSC

**Fig. 2:**
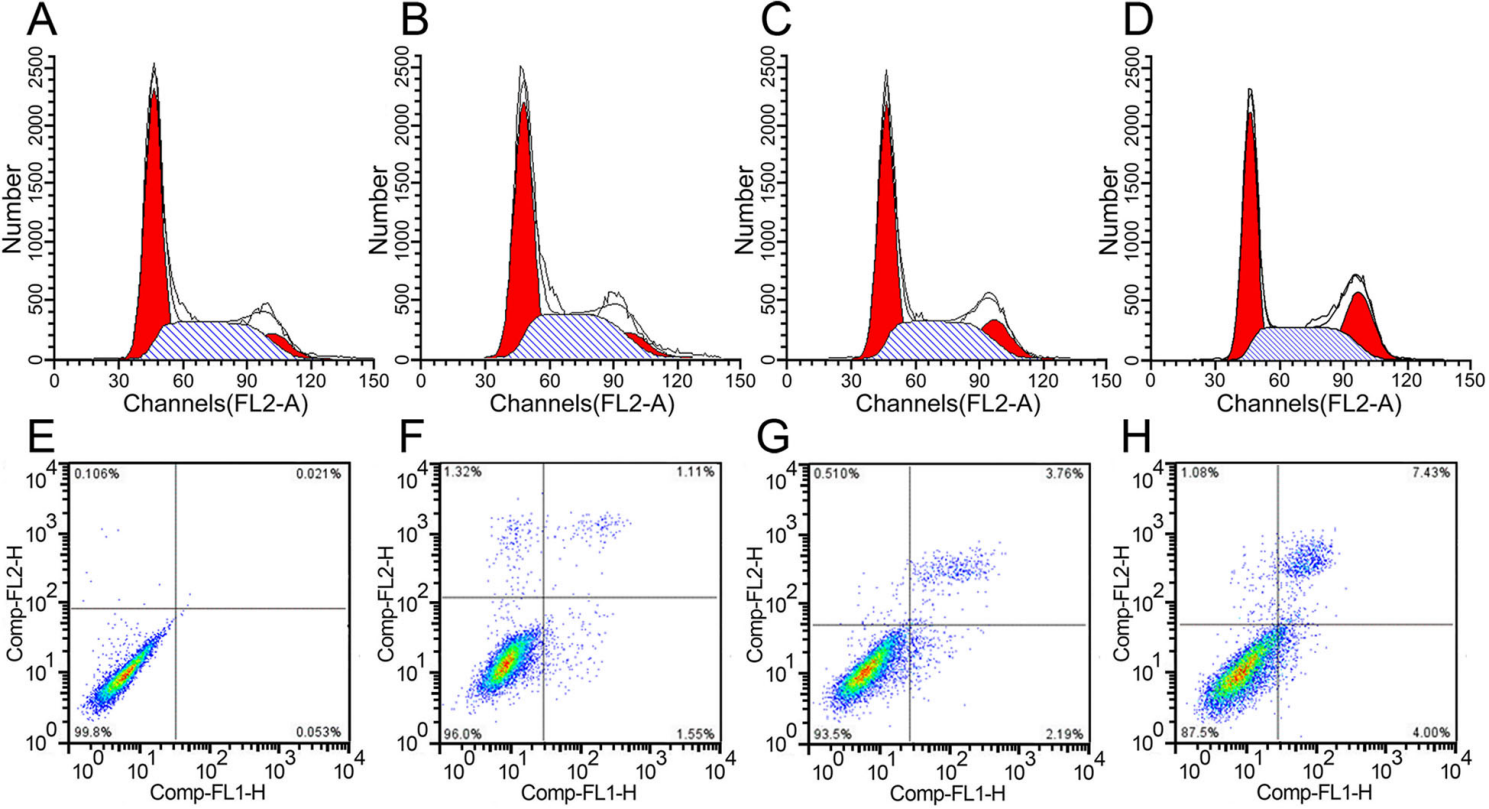
Effects of DC-CIK on the LCSC cycle and apoptosis. **a**-**d** flow cytometry analysis of the cell cycle: **a** LCSC control group; **b** LCSCs co-cultured with half density of DC-CIK cells for 24 h; **c** LCSCs co-cultured with the same density of DC-CIK cells for 24 h; **d** LCSCs co-cultured with the same density of DC-CIK cells for 48 h. **e**-**h** flow cytometry analysis of cell apoptosis measured by Annexin V-FITC antibody staining. **e** LCSC control group; **f** LCSCs co-cultured with half density of DC-CIK cells for 24 h; **g** LCSCs co-cultured with the same density of DC-CIK cells for 24 h; **h** LCSCs co-cultured with the same density of DC-CIK cell for 48 h

There was no significant difference in the G1, G2/M and S phases between phenotypically characterized human LCSC and 50% LCSC + 50% DC-CIK cells co-cultured for 48 h **(**Fig. 2b**)**. When LCSC were co-cultured with the same density of DC-CIK for 24 h, the G1 phase cells decreased by 2.93 ± 0.07%, the S phase cells by 2.13 ± 0.05% but the G2/M phase cells increased by 5.07 ± 0.15% compared to the control group **(**Fig. 2c**)**. It was noted that the LCSC appeared to be restrained in the G2/M phase (*P* < 0.05). After co-culture for 48 h, the difference increased to 8.80 ± 0.19% for the G1 phase, 4.74 ± 0.11% for the S phase, and 13.54 ± 0.48 for the G2/M phase **(**Fig. 2d**)**. Therefore, LCSC were restrained in the G2/M phase, the difference being statistically significant (*P* < 0.01).

The results for cell apoptosis demonstrated that the apoptosis rate in the control group was 0.074 ± 0.009% **(**Fig. 2e**)**. The apoptosis of LCSC co-cultured with half density DC-CIK cells for 48 h was 2.66 ± 0.15% **(**Fig. 2f**)**. There was no significant difference compared with the control group. The apoptosis of LCSC co-cultured with the same density of DC-CIK cells for 24 h was 5.95 ± 0.68% **(**Fig. 2g**)**, which was significantly different from the control group (*P* < 0.05). The apoptosis of LCSC co-cultured with the same density of DC-CIK cells for 48 h was 11.43 ± 1.04% **(**Fig. 2h**)**, again significantly different from the control group (*P* < 0.01). These results demonstrated that DC-CIK cells could significantly induce apoptosis of LCSC and obviously kill cancel stem cells.

### Effects of DC-CIK on the growth and proliferation of LCSC

**Fig. 3:**
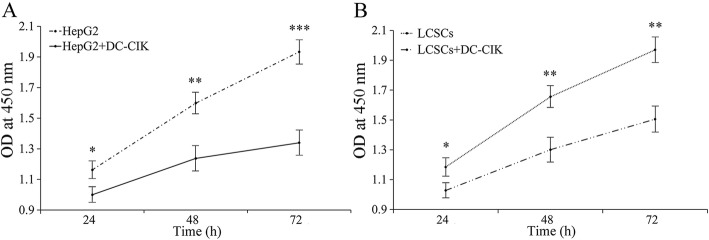
Effect of DC-CIK cells on the growth and proliferation of HepG2 and LCSCs analyzed by the CCK-8 method. **a** HepG2 cell growth rates with and without co-culture of DC-CIK cells. **b** Patients derived LCSCs growth rates with and without co-culture of DC-CIK cells. *: *P* < 0.05; **: *P* < 0.01; ***: *P* < 0.001

It was found by CCK-8 analysis that when HepG2 and LCSC were co-cultured with DC-CIKs in a 1:1 ratio, the growth rate was significantly inhibited **(**Fig. 3**)**. This finding demonstrated that LCSC growth reduction was similar to HepG2 liver cancer cells, suggesting that DC-CIK immunotherapy might also be used to kill residual cancer stem cells after chemotherapy or surgery.

### Inhibitory effects of antigen loaded DC-CIK cells on LCSC induced subcutaneous tumors in nude mice

When the first generation of LCSC were inoculated into subcutaneous tissues of nude mice at various densities (2 × 10^4^, 2 × 10^5^, 2 × 10^6^ per mouse), the tumor formation time was approximately 18 days, 12 days and 7 days, respectively.

**Fig. 4:**
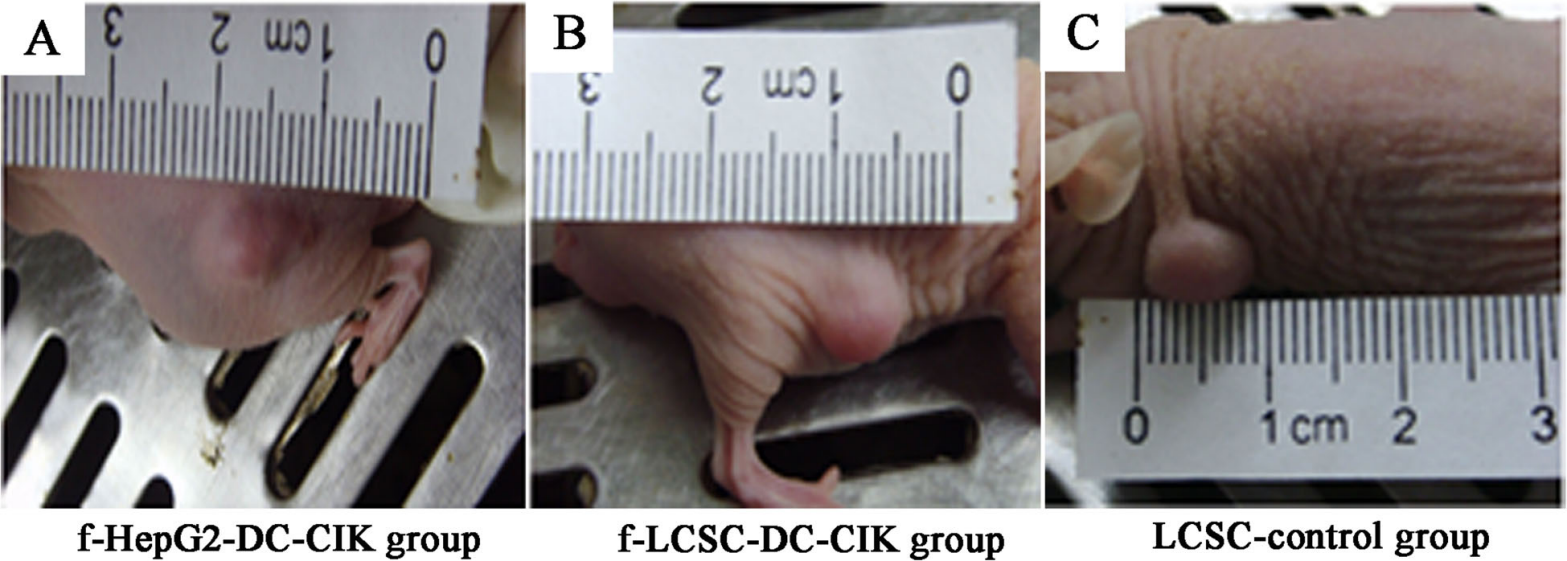
Tumor developments in nude mice 14 days after subcutaneus application of 2 × 10^6^ LCSCs. Mice with similar tumor sizes were chosen for further analyses of effects of **a** f-HepG2 DC-CIK. **b** f-LCSC DC-CIK. **c** LCSC-control experiments

**Fig. 5:**
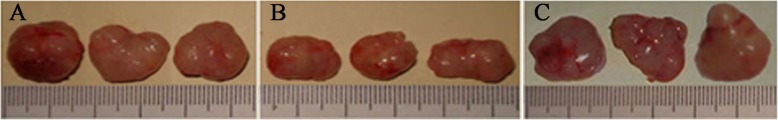
Effect of DC-CIK cell injections 14 days after tumor induction with 2 × 10^6^ LCSCs. Tumors were treated with 2 × 10^6^ DC-CIK sells, which were **a** HepG2 cell antigen loaded. **b** LCSC antigen loaded. **c** Served as the control without treatment. There were significant tumor size and weight differences in the order LCSC-control, f-HepG2-DC-CIK and f-LCSC-DC-CIK groups

Therefore, we injected 2 × 10^6^ LCSCs into nude mice allowing 14 days for tumor formation **(**Fig. 4**)**, after which 2 × 10^6^ fragmented LCSC (f-LCSC) f-LCSC-DC-CIK (*n* = 3) or 2 × 10^6^ fragmented HepG2 (f-HepG2) f-HepG2-DC-CIK (*n* = 3) antigen loaded DC-CIKs were applied to the surrounding tissues of the tumors. Tumor sizes were analyzed 14 days after the antigen loaded DC-CIK applications. There was a significant tumor reduction after f-LCSC-DC-CIK application compared to the LCSC- control and the f-HepG2 DC-CIK groups **(**Fig. 5**,** Table 1**)**.

**Table 1:** Effect of DC-CIK cell injections 14 days after tumor induction with 2 × 10^6^ LCSCs

Group	Solid tumor	Volume (mm^3^)^a^	Weight (g)^b^
A) F-HepG2-DC-CIK group	*n* = 9	202.76 ± 10.64	0.81 ± 0.09
B) f-LCSC-DC-CIK group	*n* = 9	146.35 ± 8.45	0.59 ± 0.05
C) LCSC-control group	*n* = 9	238.93 ± 11.81	1.24 ± 0.12

Note: ^a^, group A vs group B, *P* < 0.01, group A vs group C, *P* < 0.05, group B vs group C, *P* < 0.001; ^b^, group A vs group B, *P* < 0.05, group A vs group C, *P* < 0.01, group B vs group C, *P* < 0.01

### Mechanism of growth inhibition on HepG2 cell derived LCSC induced by DC-CIK

**Fig. 6:**
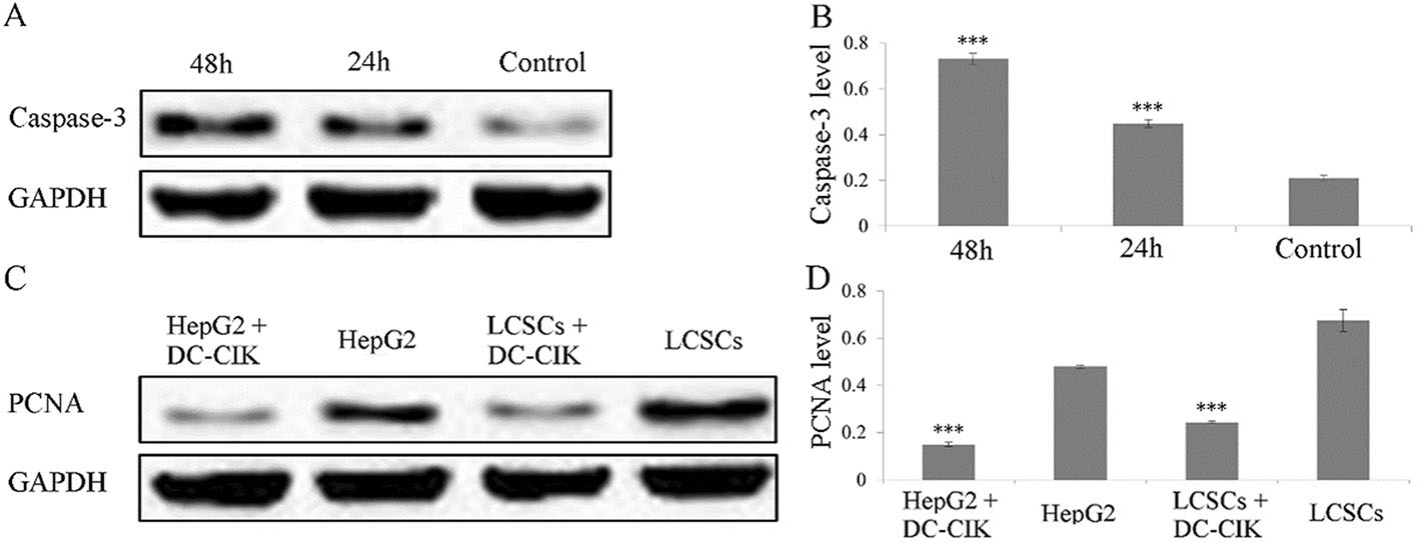
Expression analysis of caspase-3 and PCNA protein after LCSC co-incubated with DC-CIK at various times. **a** Caspase 3 expression of LCSCs after co-culture with DC-CIK cells (ratio 1:1) for 48 h, 24 h and without co-culture of DC-CIK cells (control). **b** Chart of caspase 3 expression rates with results of 3 independent western blot measurements shown in Fig. 6**a**. **c** Western Blot image of PCNA expressions from HepG2 and LCSCs cultured alone or co-cultured with DC-CIK cells for 48 h (ratio 1:1). **d** Chart of PCNA expression rates with results of 3 independent western blot measurements shown in Fig. 6c

DC-CIK could effectively inhibit the growth and proliferation of LCSC and also down-regulate the expression level of human PCNA protein. The protein expression analysis indicated that the caspase-3 antigen band (approximately 3.7 × 10^4^ molecular weight) was obvious when the same density of DC-CIK were co-cultured with LCSC for 48 h. The band intensity weakened when co-cultured for 24 h and was much weaker when DC-CIK was not co-cultured with LCSC **(**Fig. 6a and b**)**. The expressions of PCNA in HepG2 and LCSCs were significantly lower when both cell lines were co-cultured with DC-CIK cells for 48 h compared to their PCNA expressions when cultured alone **(**Fig. 6c and d**)**.

Therefore, it can be concluded that LCSC freeze-thaw antigen loaded DC-CIKs can induce LCSC generated solid tumor apoptosis, thereby reducing the volume and weights of tumors in the nude mouse model.

## Discussion

In the present study, DC-CIK was shown to inhibit the proliferation and maintenance of LCSC in vivo and in vitro. In addition, DC-CIK co-culture was found to suppress PCNA and also promote the expression level of caspase-3 protein, subsequently inducing LCSC apoptosis. Due to LCSC having a high DNA repair capacity with low pro-apoptotic protein expression levels and a general stay in the G0 phase, they were reported to be not sensitive to radiotherapy and chemotherapy [18, 19]. However, with the addition of DC-CIK cells, LCSCs became restrained to the G2/M phase. These results indicated that DC-CIK immunotherapy might be able to induce cancer stem cells to remain in the G2/M phase, which provides a new insight into restraining or eradicating cancer stem cells. PCNA, as a non-histone nucleoprotein, is expressed relative to cell proliferation, DNA synthesis, DNA repair, cell cycle regulation, chromosome recombination, DNA methylation and cell apoptosis [20, 21]. Our results demonstrated that the PCNA expression level was reduced by 33% after DC-CIK co-culture with LCSC for 48 h.

It may be concluded that DC-CIKs were able to interfere with the cancer stem cell cycle and induce caspase-3 expression, which resulted in the decline of nude mouse tumorigenic ability. However, tumor measurements have not been done over the course of treatment, which is a limitation of the present study.

Currently, tumor biotherapy (immune cells, genetic and molecular targets and stem cells) is the fourth treatment option in the clinic after surgery, radiotherapy and chemotherapy, but has promising therapeutic prospects [5, 22, 23]. Immunocell therapy is used in clinical applications and can reflect the anti-tumor characteristics of biological treatment, including tumor infiltrating lymphocytes, lymphokine-activated killer cells and CD3 monoclonal antibody activated killer cells [24, 25].

In the present study, CD3-CD56 cells were cultivated in vitro and found to have a strong recognition ability for tumor cells based on the non- major histocompatibility complex (MHC) restrictive antitumor properties of NK cells. It is noteworthy that immunosuppressant drugs, such as cyclosporine A and FK506, did not influence this ability. DC-CIK co-culture with stem cells is more effective than CIK or DC individual treatments because it can not only kill LCSC through the caspase-3 apoptosis factor pathway but also inhibit PCNA gene expression to interfere effectively with the stem cell cycle.

CIK cells are a T cell sub-population with NK cell properties but non-MHC-restricted tumor-killing activity, which have been successfully applied to various cancer patients [26]. They were reported to increase percentages of CD3+, CD4+, CD4 + CD8+, CD3 + CD56+ and NK cells and decrease.

CD8+ and regulatory T cell (Treg) subgroups [10, 27]. However, we used to treat advanced tumors patients with autogenous CIK cells but found that programmed death ligand 1 (PD-L1), lymphocyte activation gene-3 (LAG-3), T-cell immunoglobulin and mucin domain-3 (TIM-3), and carcinoembryonic antigen-related cell adhesion molecule 1 (CEACAM-1) were related to reduced anti-tumor activities during cultivation of CIK cells [28]. In addition, a factor found to be related to CIK treatment success was that patients with high expression levels of albumin had a better prognosis in comparison to patients with low expression levels of albumin [29]. Although DCs, as antigen-presenting cells (APCs), are not able to kill tumor cells directly, they can in combination with CIK cells enhance their proliferation and killing activity [30]. Huh7 HCC cancer stem cell antigen loaded DCs could activate specific endogenous cytotoxic T cell reactions against the antigen containing cells leading to the most efficient reduced tumor growth in a nude mouse model, followed by Huh7 antigen loaded and least growth inhibition by unloaded DC cell applications [31]. These results are in line with our in vitro findings, namely that DCs containing LCSC antigens are more efficient as tumor killing triggers than DCs loaded with HepG2 antibodies (Fig. 5**,** Table 1). Approaches to enhance DC-CIK activity have been to modify CIK cell activity via overexpression of IL-2 with transient plasmid transfection [32] and to overexpress IL-24 in DCs via stable transfection [11]. However, in our study we focused on combined DC-CIK applications in which DCs were loaded with LCSC specific antigens, since LCSCs are reported to be the most malignant cancer cells [6].

Currently, it remains controversial to apply DC-CIK for the treatment of tumors.

A meta-analysis of randomized controlled trials in Chinese patients concluded, that DC-CIK cell-based therapies may enhance the efficacy of chemotherapies on solid cancer but further studies without publishing bias should be performed to confirm the finding [33]. Although DC-CIK co-culture has been demonstrated to have a potent inhibitory effect on LCSC growth, much more research is needed before this therapy can be applied routinely in the clinic. Some researchers have reported that the curative effect would be better if DC-CIK immunotherapy could be applied to liver cancer tissue after treatment with chemotherapy or radiotherapy, but whether DC-CIKs can induce residual LCSC apoptosis in vivo then and thus improve the curative effect has still not been unequivocally established. This question needs to be addressed in future clinical studies. In addition, how to improve the survival rate of DC-CIK cells in vivo and maintain effective numbers in vitro is crucial to improve the clinical curative effect.

## Conclusions

DC-CIK cells could inhibit HCC and LCSC growths in vitro and in vivo with the most successful DC triggering of cell cytotoxic activity achieved by their LCSC antigen loading.

## Abbreviations

APCs: Antigen-presenting cells
bFGF: Basic fibroblast growth factor
CCK-8: Cell counting kit-8
CD: Cluster of differentiation
CEACAM-1: Carcinoembryonic antigen-related cell adhesion molecule 1
CIK: Cytokine-induced killer
CSC: Cancer stem cells
DC: Dendritic cells
EGF: Epidermal growth factor
EpCAM: Epithelial cell adhesion molecule
HCC: Hepatocellular carcinoma
IL: Interleukin
LAG-3: Lymphocyte activation gene-3
LCSC: Liver cancer stem cells
LIF: Leukemia inhibitory factor
MHC: Major histocompatibility complex
NK: Natural killer
PBMCs: Peripheral blood mononuclear cells
PCNA: Proliferating cell nuclear antigen
PD-L1: Programmed death ligand 1
rhIFN-γ: Recombinant human interferon gamma
rhIL-4: Recombinant human interleukin-4
SFM: Serum-free medium
TIM-3: T-cell immunoglobulin and mucin domain-3
TNF-α: Tumor necrosis factor α
WST-8: 2-(2-methoxy-4-nitrophenyl)-3-(4-nitrophenyl)-5- (2,4-disulfophenyl)-2H-tetrazolium monosodium salt
